# Automated tooth crown design with optimized shape and biomechanics properties

**DOI:** 10.3389/fbioe.2023.1216651

**Published:** 2023-11-28

**Authors:** Xiaoxian Jin, Shengfa Wang, Jiangbei Hu, Xiaowei Xu, Yongji Shi, Haishi Yu, Jinwu Wang, Kang Li, Xiaomin Cheng, Moyu Shao, Hongkai Wang

**Affiliations:** ^1^ School of Biomedical Engineering, Faculty of Medicine, Dalian University of Technology, Dalian, China; ^2^ School of Software Technology, Dalian University of Technology, Dalian, China; ^3^ Shandong Maier Medical Technology Co., Ltd., Rizhao, Shandong, China; ^4^ Shanghai Key Laboratory of Orthopedic Implant, Department of Orthopedic Surgery, Shanghai Ninth People’s Hospital, Shanghai Jiao Tong University School of Medicine, Shanghai, China; ^5^ West China Biomedical Big Data Center, West China Hospital, Sichuan University, Chengdu, Sichuan, China; ^6^ Shanghai Artificial Intelligence Laboratory, Shanghai, China; ^7^ Jiangsu Yunqianbai Digital Technology Co., Ltd., Xuzhou, China; ^8^ Liaoning Key Laboratory of Integrated Circuit and Biomedical Electronic System, Dalian, China

**Keywords:** full-crown restorations, statistical shape model, conditional shape model, supporting structure optimization, biomechanical simulation

## Abstract

Despite the large demand for dental restoration each year, the design of crown restorations is mainly performed via manual software operation, which is tedious and subjective. Moreover, the current design process lacks biomechanics optimization, leading to localized stress concentration and reduced working life. To tackle these challenges, we develop a fully automated algorithm for crown restoration based on deformable model fitting and biomechanical optimization. From a library of dental oral scans, a conditional shape model (CSM) is constructed to represent the inter-teeth shape correlation. By matching the CSM to the patient’s oral scan, the optimal crown shape is estimated to coincide with the surrounding teeth. Next, the crown is seamlessly integrated into the finish line of preparation via a surface warping step. Finally, porous internal supporting structures of the crown are generated to avoid excessive localized stresses. This algorithm is validated on clinical oral scan data and achieved less than 2 mm mean surface distance as compared to the manual designs of experienced human operators. The mechanical simulation was conducted to prove that the internal supporting structures lead to uniform stress distribution all over the model.

## 1 Introduction

Oral problems, including dental caries or even tooth loss, are affecting about 2.5 billion people worldwide (Peres et al., 2019; World Health Organisation, 2022). With the development of information technology, traditional manual dental crown design is gradually replaced by efficient digital design, which includes Computer-Aided Design (CAD) and Computer-Aided Manufacturing (CAM) technology (Miyazaki et al., 2009; Susic et al., 2017). In the recent decade, the continuous integration of big data and the surge of artificial intelligence has further revolutionized digital dental care, paving the way for the fully automated dental restoration design.

In the 1980s, Duret et al. (1988) first applied CAD/CAM technology to the field of dental restoration and successfully produced the first all-ceramic crown. The application of CAD/CAM simplifies the restoration design process into three parts: digital data collection, digital design of restorations, and digital processing. The creation of a database of standard teeth provides an effective method for the digital design of full-crown restorations (Cheng et al., 2009). The designer of the crown restoration adjusts the standard crown deformation to fit the patient’s dental morphology and occlusal relationship. To further improve the efficiency and accuracy of crown design, several studies related to the digital design of crown restorations have been proposed. MEHL et al. extracted surface features from 3D scan datasets, and they developed a mathematical representation model of the mandibular first molar using principal component analysis and reconstructed the surface by sparse points to test the dental inlay design task (Blanz et al., 2004; Mehl et al., 2005). Zhang (2016) used a similar strategy to develop a parametric model of the mandibular first molar and implemented the defective crown surface reconstruction task using spatially constrained feature points in the contralateral and adjacent regions of the jaw. Some researchers used a standard crown surface model to obtain an inlay model by iterative Laplace deformation with significant feature point constraints (Steinbrecher and Gerth, 2008; Jiang et al., 2016; Zhang et al., 2017) or by creating a one-to-one mapping of the standard crown model to the feature points of the retained teeth (Zheng et al., 2011) to approximate the residual region. For the automatic design task of full-crown restorations, both statistical shape model (Pascoletti et al., 2021) restorations and standard crown model deformations are included. Song et al. (Song et al., 2007) made local adjustments to standard crowns based on global and occlusal characteristic curves, but their method is only applicable to molars.

With the rapid development of deep learning theory, neural networks started to be used in dental crown restoration design. Hwang et al. (2018); Yuan et al. (2020) applied Deep Neural Networks (DNN) based on the Pix2pix (Isola et al., 2017) model to generate occlusal surfaces. Their methods are efficient but are limited to the occlusal surfaces. Tian et al. (2022) proposed a new two-stage prosthetic restoration framework to automatically reconstruct functional occlusal surfaces with realistic details. Lessard et al. (2022) improved PF-Net (Huang et al., 2020) by using a point cloud-completing method to accomplish the tooth completion task. However, this study did not consider the morphology of the inner surface of the prepared tooth to obtain a complete crown.

In summary, the existing algorithms have significantly automated the design of crown restorations. However, it is still challenging to preserve anatomical morphological features such as developmental grooves and crests. Some of the studies only focus on occlusal surface reconstruction without close matching of the restoration with the adjacent teeth and the preparation, leaving the design process incomplete for clinical applications. Moreover, most existing studies lack biomechanical property optimization, potentially leading to localized stress concentration which reduces the working life.

To solve the above problems, an automatic full-crown restoration algorithm is proposed in this paper. The main contributions of this paper are as follows:

(1) A parametric statistical shape model (SSM) of the tooth was constructed based on a clinical dental scan data library which preserved the natural morphological characteristics of the occlusal surface. This model was deformed and matched to the patient’s oral scan to achieve an automatic crown design.
(2) Different from the existing studies which only use tooth SSMs, a conditional shape model (CSM) was constructed in this work to model the occlusal and adjacency relationships between neighboring teeth, ensuring proper inter-tooth shape dependencies of the designed crown.
(3) In addition to the crown body design, an automatic process was developed to closely fit the deformable crown to the preparation finish line, resulting in a crown model ready for subsequent CAM.
(4) The internal support structure was optimized to prolong the working life and save the additive manufacturing material. The biomechanical simulation was performed to verify that the optimized support structure led to uniform stress distribution in all parts of the crown.

## 2 Materials and methods

### 2.1 Technical process

**FIGURE 1:**
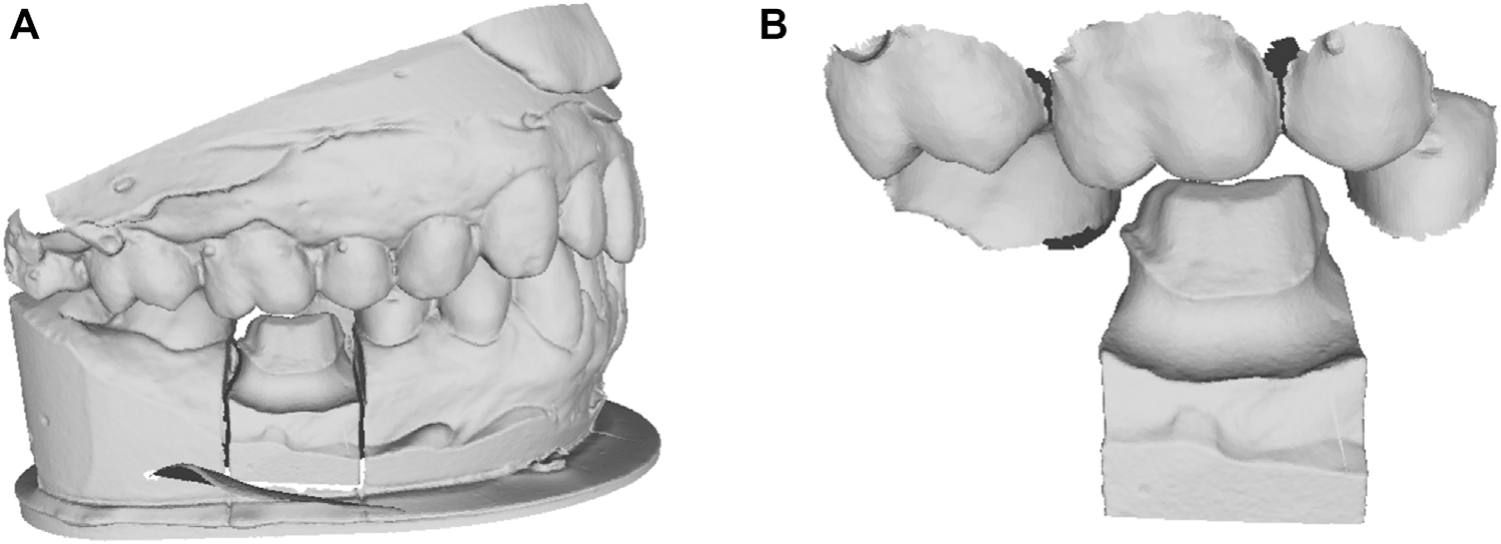
The clinical dental scan data. **(A)** the oral scan model with missing tooth; **(B)** the cropped local adjacent teeth and preparation model.

This work addresses the problem of missing tooth crown design using the existing adjacent teeth as shape references, which are shown in Figure 1.

**FIGURE 2:**
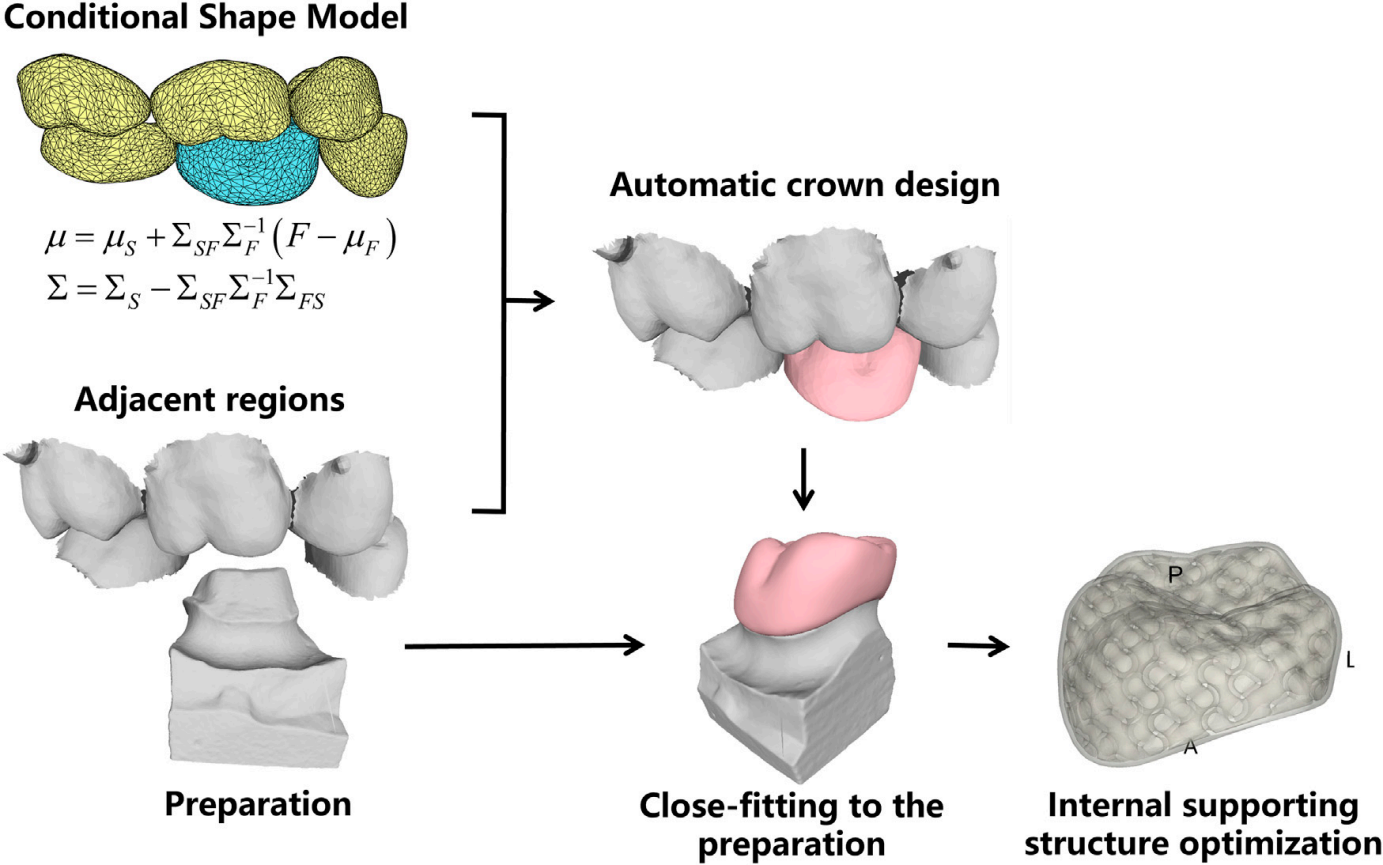
Technical process.

Figure 2 shows the technical process of the proposed algorithm, which mainly consists of the following steps:

(1) Conditional shape model (CSM) construction. Based on a library of clinical oral scan data, the deformable statistical shape models (SSMs) of different teeth were constructed. After that, the inter-teeth shape correlation between adjacent SSMs was modeled using the CSM approach.
(2) Shape model matching with patient oral scan. First, the SSM of the neighboring existing teeth is matched to the oral scan. Then the missing crown model was created using the CSM, and the possible intersections between the crown and its neighboring teeth are automatically removed to ensure reasonable physical contact.
(3) Crown fitting to the preparation. The preparation finish line is automatically identified and the bottom of the crown is deformed to closely fit to the finish line.
(4) Internal supporting structure optimization. The porosity and direction of the internal supporting structure were optimized to ensure uniform stress distribution over the entire crown for the sake of prolonged working life and efficient material consumption.

### 2.2 Statistical shape model construction

SSM (Cootes et al., 1995) parametrically represents the average shape of a class of 3D models and their shape variations, as defined by the following equation,

$$X=\bar{X}+\sum_{i=1}^{p}a_{i}\Phi_{i},$$ (1)

where, X is the statistical shape model, $\bar{X}$ is the average model, $\Phi_{i}$ represents the different deformation modes obtained from the principal component analysis (PCA), p is the number of the deformation modes, and $a_{i}$ represents the shape coefficients of the different deformation modes.

**FIGURE 3:**
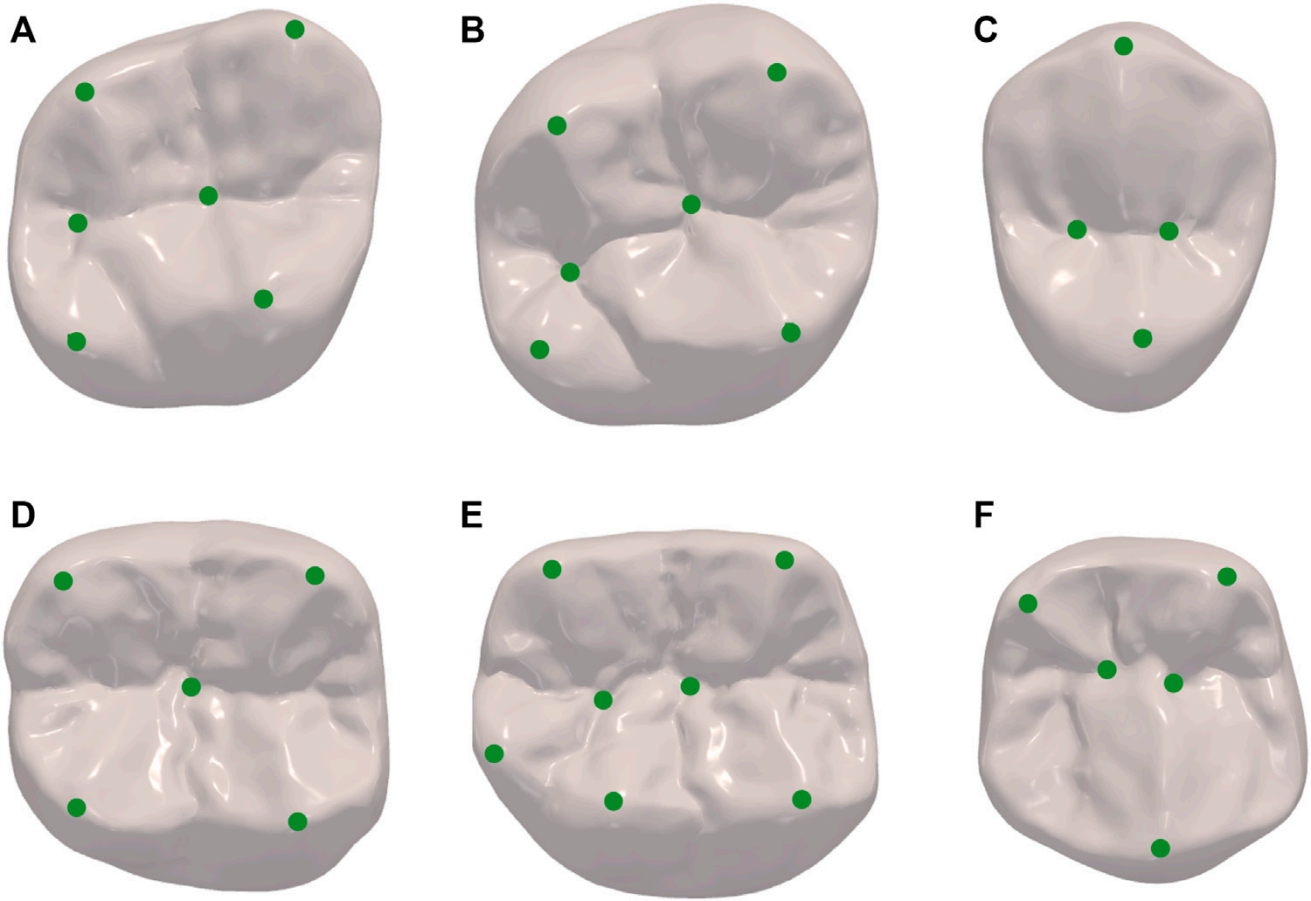
Landmarks of different dental positions indicating the location of prominent features such as on the grooves, pits and cusps. **(A)** maxillary second molar; **(B)** maxillary first molar; **(C)** maxillary second premolar; **(D)** mandibular second molar; **(E)** mandibular first molar; **(F)** mandibular second premolar.

To construct the SSM of dental crown, a standard template was aligned to all other clinic oral scan models using the Thin Plate Spline Robust Point Matching (TPS-RPM) (Chui and Rangarajan, 2003) method, to establish the surface vertices correspondence of different templates. In addition, to restrict the vertex distribution, we selected prominent feature landmarks on the occlusal surface of the crown, such as on the grooves, pits, and cusps, based on the anatomical characteristics of different dental positions. These landmarks were used to guide the deformation of the standard template. Figure 3 shows the landmarks selected for different dental positions, where Figures 3A–F are respectively the right maxillary second molar, right maxillary first molar, right maxillary second premolar, right mandibular second molar, right mandibular first molar, and right mandibular second premolar.

From this, we can obtain a set of ideal data, each mesh has the same number of vertices, and the corresponding vertices in different meshes are located in the same anatomical position. N vertices of each mesh can form a vector x ,

$$x={\left[x_{1},y_{1},z_{1},...,x_{n},y_{n},z_{n}\right]}^{T},$$ (2)

Where $\left(x_{j},y_{j},z_{j}\right)$ is the coordinate of the j -th vertex in the mesh.

To cancel the inter-subject variations in translation, rotation, and scaling, we used Generalized Procrustes analysis (GPA) (Bookstein, 1996) for the sample vectors. Next, we statistically model the aligned shape vectors by principal component analysis (PCA). For the shape vectors represented by Eq. (2), the average model can be obtained by simply averaging the samples as Eq. (3).

$$\bar{X}=\frac{1}{k}\sum_{m=1}^{k}x_{m},$$ (3)

where k is the number of mesh used to set up the SSM.

The singular value decomposition (SVD) of Eq. (4) can be easily calculated to obtain the eigenvectors $\Phi_{i}$ and the corresponding eigenvalues $\lambda_{i}$ , namely, the different modes of deformation and their corresponding variances, with the largest eigenvalue corresponding to the most significant mode of variation.

$$L=\left(\left(x_{1}-\bar{X}\right)...\left(x_{k}-\bar{X}\right)\right),$$ (4)

where k is the number of mesh used to set up the SSM.

The parameter $a_{i}$ in Eq. (1) is used to control the shape deformation, which is usually controlled within the range of $\left[-3\sqrt{\lambda_{i}},3\sqrt{\lambda_{i}}\right]$ to ensure that the deformation is reasonable.

### 2.3 Conditional shape model construction

According to the proposed method, the SSMs are constructed for the missing tooth (described as “son model” in the following) and its adjacent teeth as a whole (described as “father model” in the following). Since the teeth perform important masticatory functions, the size and position of the missing crown mainly depend on its adjacent teeth. In other words, there is a high correlation between the shape coefficients of the two. We parametrically describe this relationship by building the conditional shape model (CSM) (Iglesias and de Bruijne, 2007).

The coefficients of the SSM of the son model S depend heavily on the father model F . The distribution of S can be modeled as a conditional Gaussian distribution $P\left(S\left|F\right.\right)$ under the condition that F is known,

$$P\left(S\left|F\right.\right)=N\left(\mu ,\Sigma \right),$$ (5)

Representations of μ and Σ can be derived according to (Iglesias and de Bruijne, 2007):

$$\mu =\mu_{S}+\Sigma_{SF}\Sigma_{F}^{-1}\left(F-\mu_{F}\right),$$ (6)

$$\Sigma =\Sigma_{S}-\Sigma_{SF}\Sigma_{F}^{-1}\Sigma_{FS},$$ (7)

where, μ is the conditional mean, Σ is the conditional covariance, $\mu_{S}$ and $\mu_{F}$ are the average shape coefficients of S and F respectively, $\Sigma_{S}$ and $\Sigma_{F}$ are the variances of S and F respectively, and $\Sigma_{SF}$ and $\Sigma_{FS}$ are the covariance matrices of both.

When a set of SSM distribution coefficients of the father model F is given, the SSM distribution coefficients of the son model S can be obtained according to the above probability model, namely, the son model matching the shape of the father model is obtained.

### 2.4 SSM matching

After constructing the deformable models, in practical application, to obtain the set of shape coefficients of the father model, it is necessary to match the SSM of the father model to the patient’s oral scan model.

According to Eq. (1), the SSM of the father model can be expressed as follows,

$$F=\bar{F}+\sum_{i=1}^{p}a_{i}^{F}\Phi_{i}^{F},$$ (8)

The average model vertices of the father model can form a vector $\bar{F}={\left[x_{1}^{F},y_{1}^{F},z_{1}^{F},...,x_{n}^{F},y_{n}^{F},z_{n}^{F}\right]}^{T}$ , where $\left(x_{j}^{F},y_{j}^{F},z_{j}^{F}\right)$ is the coordinate of the j -th vertex in the mesh.

To improve the computational efficiency, the Iterative Closest Point (ICP) algorithm (Besl and McKay, 1992) was used to initially align the father model with the patient’s oral scan model. The nearest points of $\bar{F}$ in the patient oral scan model were searched during each iteration and constituted the vector $P={\left[x_{1}^{P},y_{1}^{P},z_{1}^{P},...,x_{n}^{P},y_{n}^{P},z_{n}^{P}\right]}^{T}$ .

The shape coefficients of the father model $a_{F}={\left[a_{1}^{F},a_{2}^{F},...,a_{k}^{F}\right]}^{T}$ are computed by solving the linear system to minimize the least squares error,

$$\min{\left\|P-\bar{F}-\sum_{i=1}^{p}a_{i}^{F}\Phi_{i}^{F}\right\|}_{2}^{2},$$ (9)

According to Eq. (6), the shape coefficient of the son model can be expressed as follows,

$$a_{S}=\mu_{S}+\Sigma_{SF}\Sigma_{F}^{-1}\left(a_{F}-\mu_{F}\right),$$ (10)

Then the results of the father model aligned to the patient’s oral scan model can be obtained as follows,

$$F^{\prime }=\bar{F}+\sum_{i=1}^{p}a_{i}^{F}\Phi_{i}^{F},$$ (11)

The son model matching with the shape of the father model,

$$S^{\prime }=\bar{S}+\sum_{i=1}^{p}a_{i}^{S}\Phi_{i}^{S},$$ (12)

To further improve the alignment accuracy, the TPS-RPM alignment algorithm was used to align $F^{\prime }$ with the patient’s oral scan model again. Since the ICP alignment had matched the two coarsely, TPS-RPM converged at a fast rate to obtain the transformation matrix, which was applied to $S^{\prime }$ .

Finally, the intersection-removing algorithm was used to ensure that there was no unreasonable intersection between the crown and its adjacent teeth. Specifically, the vertices of the crown were looped through, and those located inside the adjacent teeth were moved outward along the direction of the normal vector until all vertices did not need to be adjusted. Finally, the face intersection was detected to ensure reasonable contact between the crown and its adjacent teeth.

### 2.5 Preparation fitting

**FIGURE 4:**
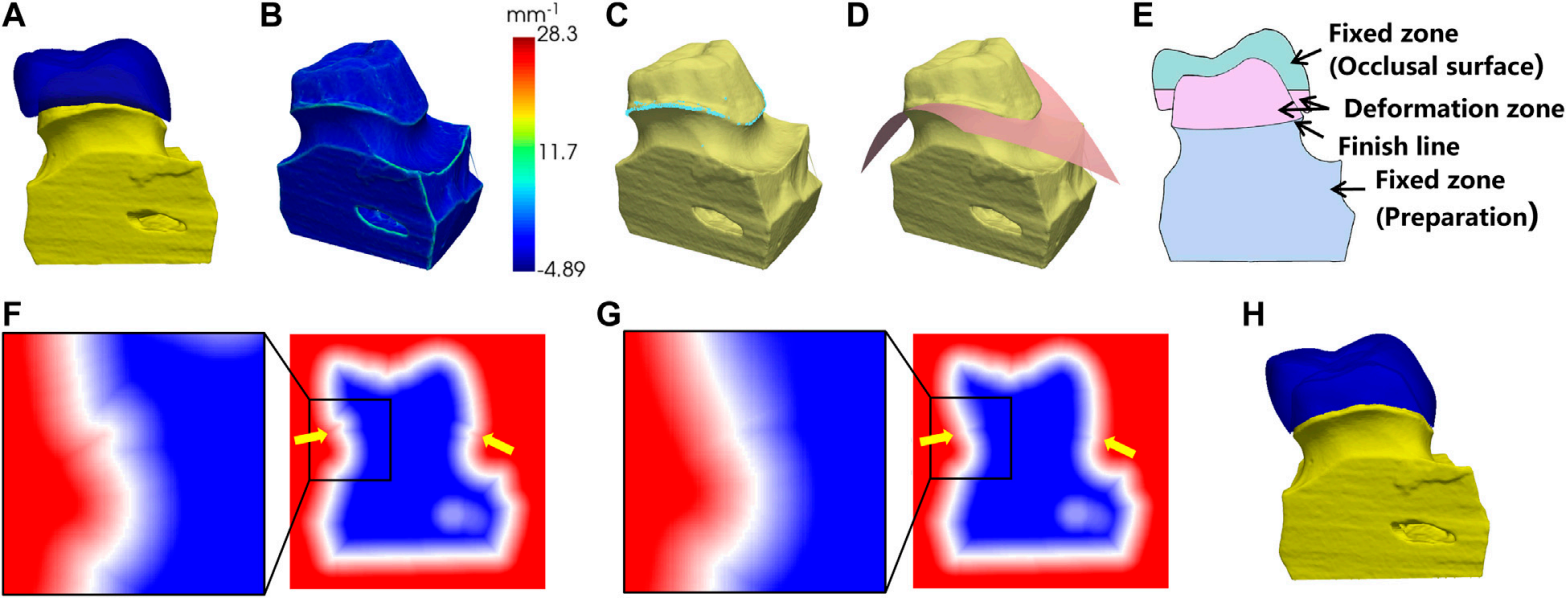
Preparation fitting process. **(A)** the crown before the fitting; **(B)** surface curvature of the preparation; **(C)** points on the finish line (in light blue color); **(D)** fitting the cross surface of the finish line; **(E)** the cross-sectional view of **(A)**; **(F)** the SDF before fitting; **(G)** the SDF after fitting; **(H)** the fitting result.

The above steps ensure a clear occlusal surface with anatomical features and a harmonious external surface with the morphology of its adjacent teeth (Figure 4A). However, for practical clinical usage, the bottom of the crown must be closely fitted to the preparation.

As shown in Figure 4B, we computed the local curvature for the surface vertices on the preparation model. The vertices with maximum local curvature on the upper part of the model were selected as the candidate points of the finish line (Figure 4C). Next, a polynomial surface is fitted to the candidate points and the crown is cut by this surface to remove the extra parts below the finish line (Figure 4D).

To fit the bottom of the crown to the finish line of preparation, the sign distance function (SDF) was used to control the crown deformation. The SDF is defined as the distance from a point in space to the surface with a positive interior and negative exterior, and the surface is expressed as the 0-equivalent surface of the SDF. First, the mesh of the crown and the preparation were converted into 3D voxels separately and combined into one whole. The SDF values at the vertices of the voxels were calculated, as shown in Figure 4F. In order to create a smooth transition surface from the bottom of the crown to the preparation, the calculated SDF array was diffusion filtered. The cross-sectional view of the crown and the preparation is shown in Figure 4E. To ensure that the shape of the occlusal surface and the preparation below the finish line were maintained, the SDF values in the fixed zone were fixed during the diffusion, while the SDF values in the deformation zone were gradually smoothly and contracted to the finish line. The SDF values after diffusion were shown in Figure 4G. Finally, the 0-equivalent surface of the SDF was converted into a triangular mesh using the marching cube algorithm, resulting in a closed crown that matched the shape of the preparation, as shown in Figure 4H.

### 2.6 Internal supporting structure optimization

Nowadays, additive manufacturing (AM) is increasingly used in the CAM of the dental crown. Compared to the traditional computer numerical control (CNC) technique, AM is more flexible for producing complex structures thus facilitating the manufacturing of complicated support structures inside the object. In this work, skeletonized internal supporting structures of the crown are designed to improve the biomechanical properties and to save the materials for additive manufacturing.

The supporting structure is designed using the triply periodic minimal surfaces (TPMS) method that we previously proposed for generating porous structures (Wang et al., 2022). To effectively represent the porous structures, the period function and the wall-thickness function, which control parameters continuously, were created to control the number of holes (topology change) and the thickness of the structure wall (geometry change), respectively. These two continuous functions, which directly compute integrals and gradients, converted the mechanical problem with given boundary conditions into a computable mathematical model. Moreover, the proposed optimization can be implemented directly on the function representation, only one time of voxel partition is needed to generate the finite elements for integral calculations, instead of multiple times of remeshing of the iteratively updated model. The main optimization process consisted of two steps, where the period function was optimized as a coarse adjustment and the wall-thickness function was used for fine optimization. This two-step optimization can also be accelerated using a global-local radial basis interpolation strategy, which efficiently minimized the compliance of the structures. Finally, a porous structure was obtained with uniform stress distribution.

Specifically, firstly, the initial TPMS-based structure is obtained according to the input crown model and the loading conditions. The initial porosity is 60%, and the wall thickness is set within [0.2, 1.0] mm. Note that the parameters can be adjusted according to the applications. Secondly, a function representation-based optimization formulation is defined to minimize the compliance of the porous shell structure. The bottom of the crown is rigidly fixed to prevent any lateral movement, while disregarding the frictional forces. Additionally, we impose the constraint of vertical pressure exclusively on the occlusal surface of the crown. Thirdly, the aforementioned two-step optimization strategy is exploited to optimize the topology and the geometry, respectively. Finally, we obtain the optimized porous structure of the crown with continuous geometry changes and smooth topology changes that have reasonable stress distribution. In the final processing, an outer shell is added to wrap the porous structures. The default thickness of the shell is set to 0.6–1 mm according to the precision of 3D printers.

## 3 Results and discussion

To verify the proposed method, we choose the first molars as the missing teeth for crown design, since the first molars have the highest damage rate according to the statistical surveys (Zheng et al., 2011; Jiang et al., 2016; Zhang et al., 2017). Twenty representative samples of different shape patterns were used to construct the SSMs and CSM. In the subsequent sections, we will demonstrate the results for crown SSM and CSM construction through free deformation experiments, and conduct tests on clinical data to quantify the design accuracy. The internal supporting structure was optimized to result in uniform stress distribution, which was proved through biomechanical simulation.

### 3.1 SSM construction results

**FIGURE 5:**
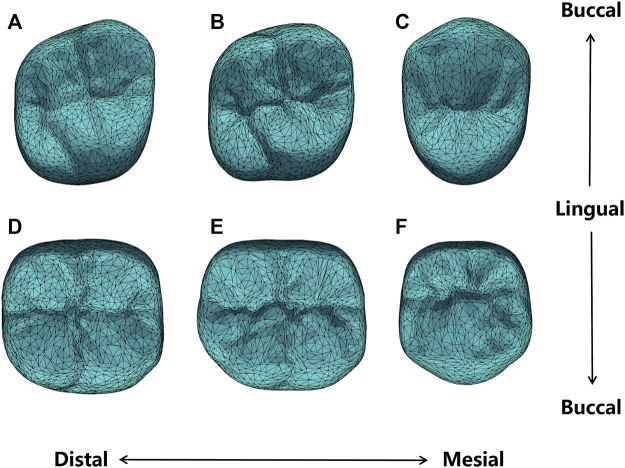
Average shape models of **(A)** maxillary second molar; **(B)** maxillary first molar; **(C)** maxillary second premolar; **(D)** mandibular second molar; **(E)** mandibular first molar; **(F)** mandibular second premolar.

Previous to the automatic crown design, the SSMs of the target crown (for the first molars) and its adjacent teeth are constructed. Figure 5 shows the average shape models of the SSMs, where (A-F) are respectively the maxillary second molar, maxillary first molar, maxillary second premolar, mandibular second molar, mandibular first molar, and mandibular second premolar. Figure 5 only shows the models of the right side teeth, whereas the left side teeth models are similar.

It can be seen from Figure 5 that the average shape models maintained the anatomical details of the occlusal surface. Specifically, the maxillary second premolar buccal cusp is similar in height to the lingual cusp, while the mandibular second premolar appears round. The maxillary first molar and the second molar are rhomboidal and generally have four cusps, of which the mesialingual cusp has the largest area as the main functional cusp, but the latter presents a more pronounced rhomboidal shape and a slightly narrower crown. The mandibular first molars typically have five cusps, while the mandibular second molars are cross-shaped. All these detailed features are presented in our shape model.

**FIGURE 6:**
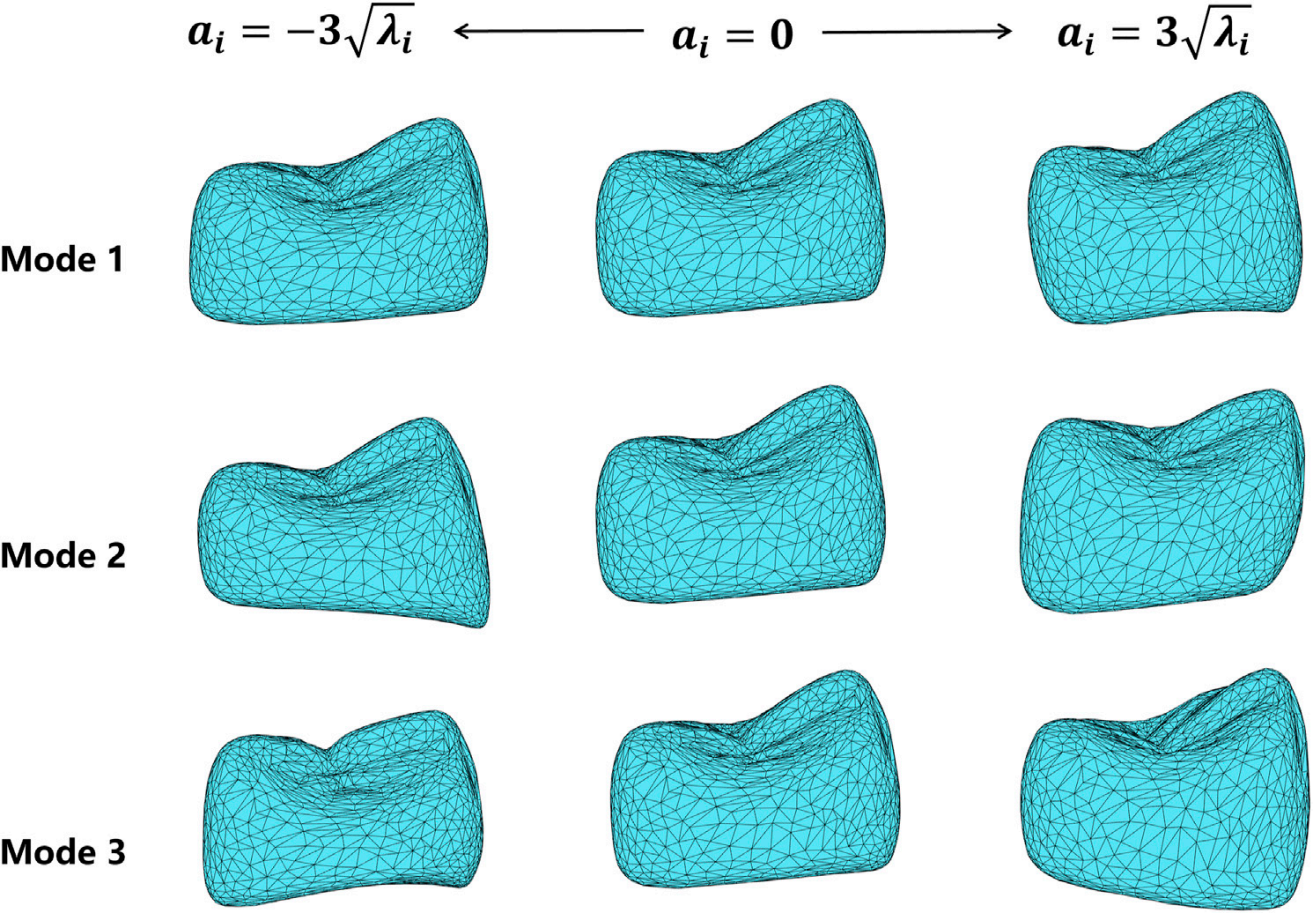
Deformation results of the first three shape variation modes, varying in the range of $\alpha_{i}\in \left[-3\sqrt{\lambda_{i}},3\sqrt{\lambda_{i}}\right]$ , where i represents the mode index. Each row shows the deformation effect of one mode, and the middle column shows the average model.

To observe the shape variation modeling ability of the SSM, different shape coefficients were adjusted to produce deformed shape instances. Figure 6 shows the deformation effects of the first three shape modes. Mode 1 demonstrates the adjustment of width and height aspect ratios. Mode 2 shows the height variation of the lingual and buccal sides of the crown bottom, which is to adapt to the gingival curvature of different patients. Mode 3 controls the relative height changes of the mesial and distal crown and the crest of the tooth.

### 3.2 CSM construction results

To construct the CSM, we take the mandibular first molar SSM as the son model, and its five neighboring teeth as a whole SSM as the father model. Then the dependence between the shape coefficients of the two SSMs was constructed as a CSM.

**FIGURE 7:**
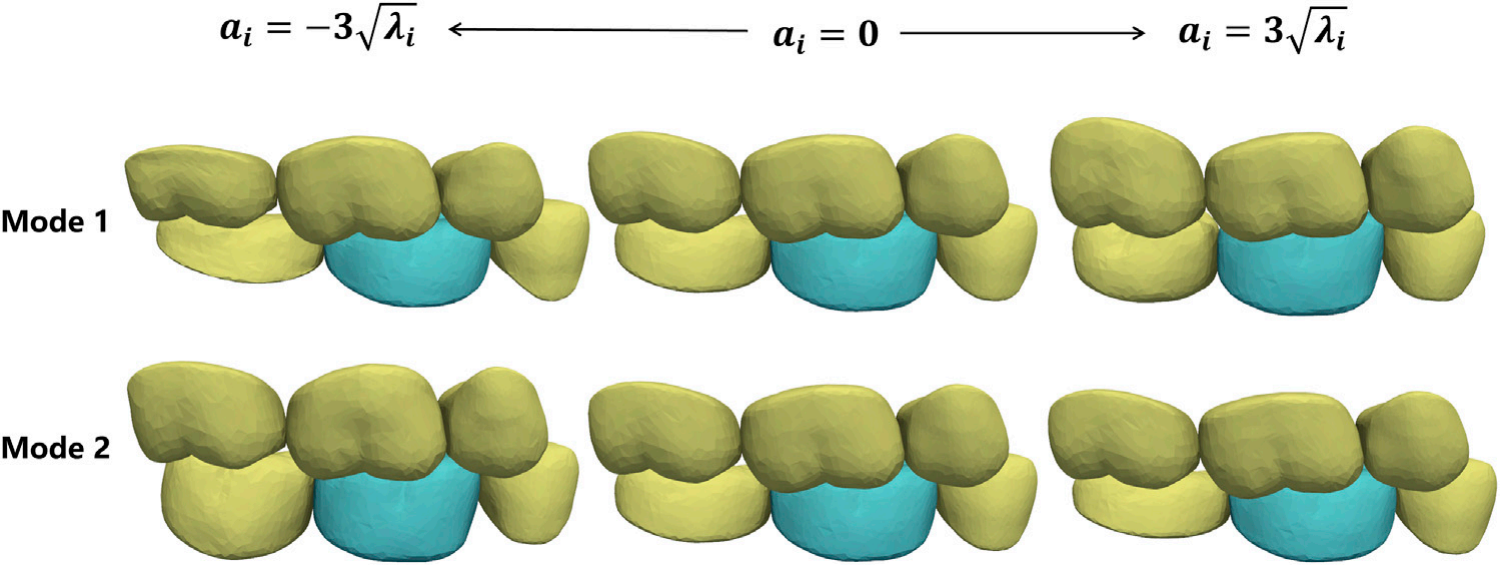
Shape deformation of the target crown (green) following the shape changes in its adjacent teeth (yellow). Each row shows the active deformation effect of the father model in one mode and the corresponding son model generated using the CSM.

To verify the constructed CSM, we actively changed the shape coefficients of the father model and generated the corresponding son model using CSM. Figure 7 shows the son model shape adaptation following the father model change under the first two modes. Mode 1 mainly focuses on the adjustment of the occlusal position of the father model, and mode 2 mainly shows the change in the tooth height. It can be seen that the son model follows well with the deforming father model in both position and size variations.

Similarly, the CSM of the maxillary first molar was established.

### 3.3 Automatic crown design results

The proposed crown design method was used to test 20 oral scan data randomly selected from our database of maxillary first molar and mandibular first molar, respectively. All the test data were not included in the model training dataset. The first molar and its five adjacent teeth are manually cropped from the organ scan for algorithm validation.

**FIGURE 8:**
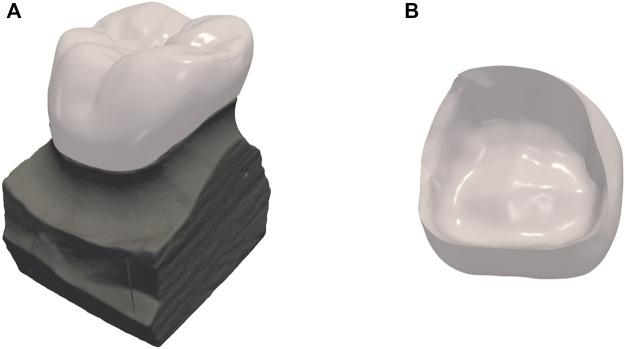
Results of dental crown design. **(A)** the crown design overlaid on top of the preparation; **(B)** the internal surface of the crown.

Figure 8A shows the crown design of one representative test data, with an inner surface that matches the shape of the preparation as shown in Figure 8B.

To quantify the accuracy of crown design, we used the manually designed crown by experienced designers as the reference standard. The Chamfer Distance (CD) between our result model and the expert designed to quantitatively assess the accuracy of the SSM matching,

$$CD\left(P_{1},P_{2}\right)=\frac{1}{\left|P_{1}\right|}\sum_{x\in P_{1}}\min_{y\in P_{2}}\left\|x\right.{\left.-y\right\|}^{2}+\frac{1}{\left|P_{2}\right|}\sum_{y\in P_{2}}\min_{x\in P_{1}}\left\|y\right.{\left.-x\right\|}^{2}$$ (13)

where, $P_{1}$ is the point set consisting of the vertices of the crown design, $P_{2}$ is the point set consisting of the vertices of the manually designed crown, x and y are the coordinates of the vertices in $P_{1}$ and $P_{2}$ respectively.

**FIGURE 9:**
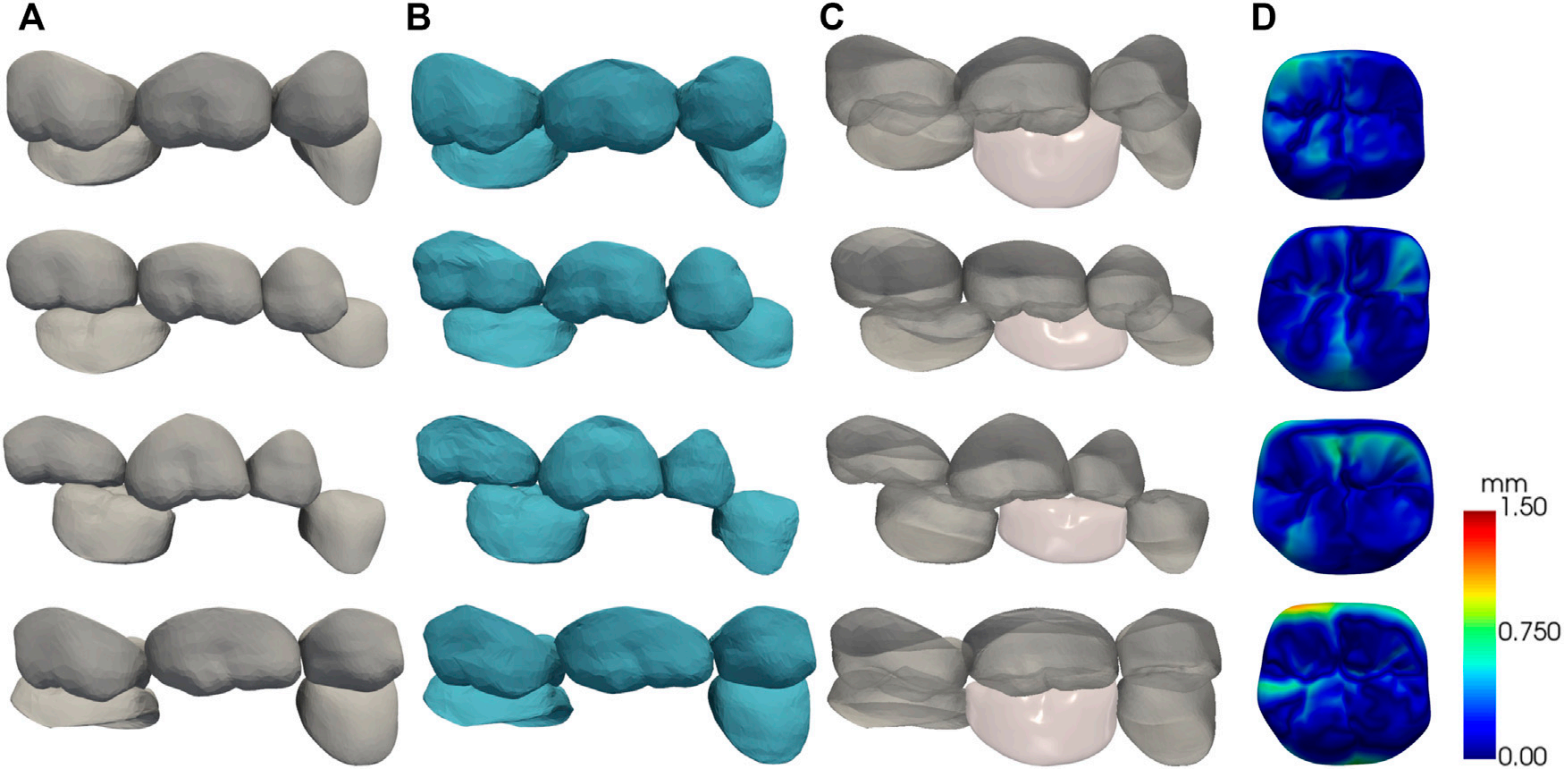
The results of the automatic crown design, each row shows one of the test data. **(A)** test data; **(B)** SSM matching results; **(C)** crown design results; **(D)** surface CD map shown in pseudo color.

A randomly selected part of the test results is shown in Figure 9. Each column from left to right shows the test data, the deformation results after matching the father model to the test data, the automatic design results of the crown, and the visualization of crown design quality based on CD. For visual assessment, the crown designed by our method has consistent size with the adjacent teeth and complements the surrounding dentition without noticeable gaps or overlaps.

The quantitative test results are shown in Table 1. The American Board of Orthodontics (ABO) accepts crown designs with errors within 0.5 mm (Casko et al., 1998) as a criterion for quality evaluation. In our clinical data experiment, we observed CD of 0.26±0.08 mm for the maxillary first molar and 0.28±0.07 mm for the mandibular first molar.

**TABLE 1:** The quantitative test results.

	Distance (mm)	
	Maxillary first molar	Mandibular first molar
CD	0.26 ± 0.08	0.28 ± 0.07
Mean	0.26 ± 0.07	0.26 ± 0.06
Median	0.22 ± 0.07	0.21 ± 0.06
Max	0.96 ± 0.29	1.11 ± 0.45
Min	(0.17 ± 0.06)e^−2^	(0.15 ± 0.06)e^−2^

### 3.4 Internal supporting structure optimization results

**FIGURE 10:**
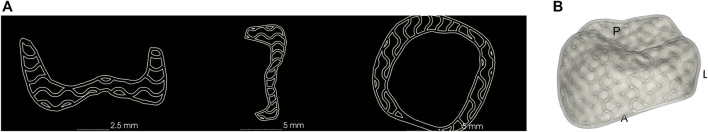
Internal supporting structure design of the crown restoration. **(A)** 2D coronal, sagittal and axial section slices of crown model showing the internal supporting structure; **(B)** 3D transparent rendering showing the internal supporting structure.

The internal supporting structure of the crown was optimized using the proposed method. The obtained internal porous structure is shown in Figures 10A, B. The optimized porosity is around 70%, and the wall thickness is adjusted within [0.2, 0.6] mm.

**FIGURE 11:**
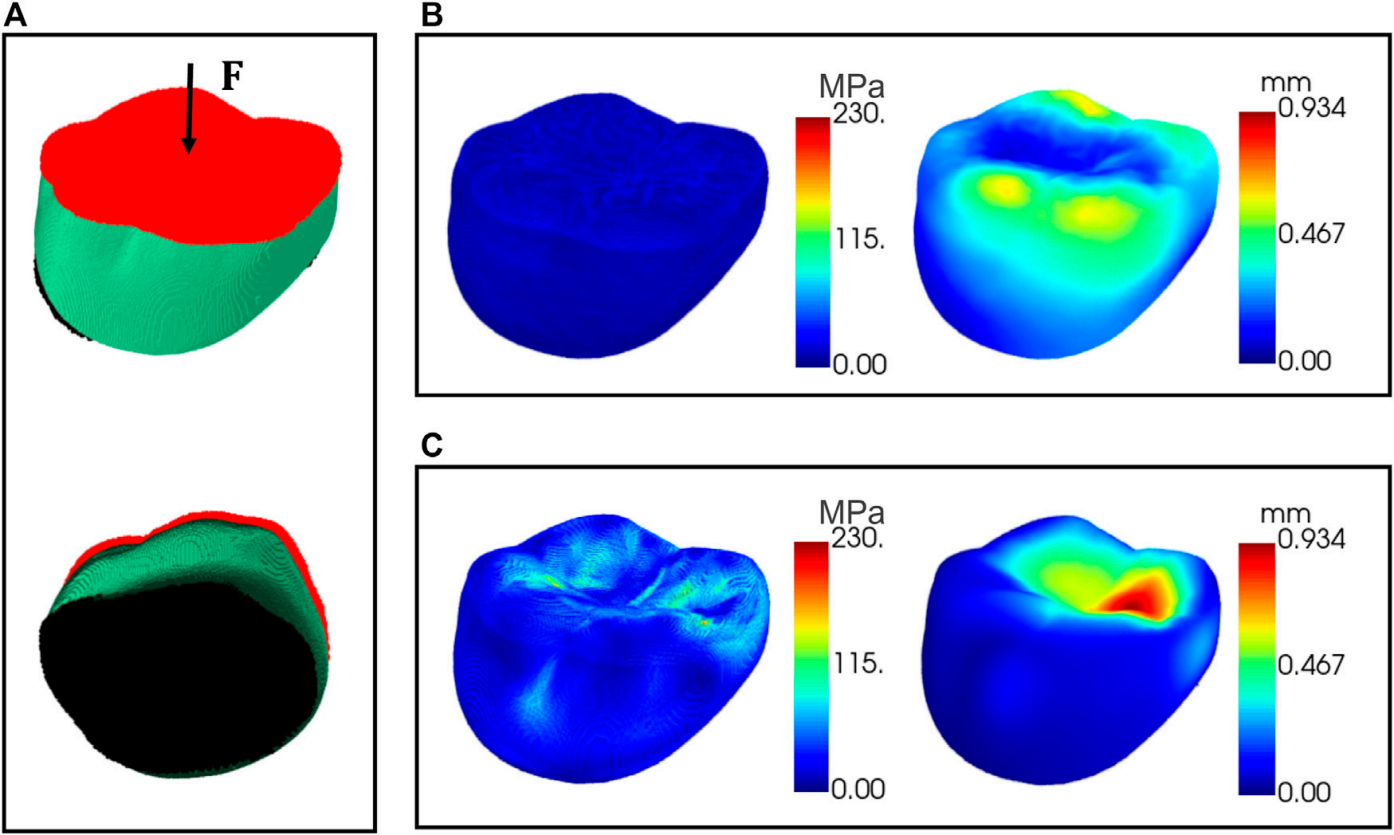
Finite element simulations of the crown. **(A)** The area applied with external pressure (red) and the area of fixed support (black); **(B)** displacement and stress maps for the crown with porous internal structure; **(C)** displacement and stress maps for the solid model.

To verify the biomechanical property of the optimized internal supporting structure, finite element simulations were conducted for the designed crown with porous internal structures. The crown material was assumed to be nickel-chromium alloy, with Young’s modulus 214 GPa and Poisson’s ratio 0.3. A nodal load of 0.5 N was applied to the occlusal surface with fixed support conditions to the bottom surface of the model as shown in Figure 11A. Figure 11B shows the distribution maps of the deformation displacement and stress for a representative test data under the given boundary conditions. It can be seen that the stress is uniformly distributed at all internal locations of the crown and the displacement is more concentrated in the cusp region. This result is compared to the simulation of the solid model under the same boundary conditions. The resultant distribution maps of displacement and stress are demonstrated in Figure 11C. The optimized porous internal structure diffused the external pressure and resulted in a more uniform stress distribution than the solid model, and the material used is also saved due to the porous architecture.

## 4 Conclusion

In this paper, we propose an automatic design algorithm for crown restoration by matching the conditional shape model of the target crown and its adjacent teeth. The missing crown with realistic occlusal surface morphology was automatically generated and fit to the finish line of the preparation. The geometry of the internal supporting structure was optimized to yield uniform stress distribution. As a proof of concept, our biomechanical simulation proves that the porous optimization resulted even stress and strain distribution over the entire crown. We will further validate this advantage with real crown mechanical test in future study. In future work, we will continue to expand the data sample library and automate the identification of missing tooth positions since this method still relies on manual specification of the target teeth location.

## Data Availability

The datasets presented in this article are not readily available because the datasets used during the current study are available from the corresponding author upon reasonable request. Requests to access the datasets should be directed to biomedimg@163.com.

## References

[B1] Besl P. J. McKay N. D. (1992). A method for registration of 3-D shapes. IEEE Trans. Pattern Analysis Mach. Intell. 14, 239–256. 10.1109/34.121791

[B2] Blanz V. Mehl A. Vetter T. Seidel H.-P. (2004). “A statistical method for robust 3D surface reconstruction from sparse data,” in Proceedings. 2nd International Symposium on 3D Data Processing, Visualization and Transmission, 2004. 3DPVT 2004, Thessaloniki, Greece, 6-9 September 2004, 293–300. 10.1109/TDPVT.2004.1335212

[B3] Bookstein F. L. (1996). Landmark methods for forms without landmarks: localizing group differences in outline shape. Proc. Workshop Math. Methods Biomed. Image Analysis, 279–289. 10.1109/MMBIA.1996.534080 9873908

[B4] Casko J. S. Vaden J. L. Kokich V. G. Damone J. James R. D. Cangialosi T. J. (1998). Objective grading system for dental casts and panoramic radiographs. Am. J. Orthod. Dentofac. Orthop. 114, 589–599. 10.1016/s0889-5406(98)70179-9 9810056

[B5] Cheng X. An T. Liao W. Dai N. Yu Q. Lu P. (2009). Establishment of database with standard 3D tooth crowns based on 3DS MAX. Sheng Wu Yi Xue Gong Cheng Xue Za Zhi 26, 866–868. 19813628

[B6] Chui H. Rangarajan A. (2003). A new point matching algorithm for non-rigid registration. Comput. Vis. Image Underst. 89, 114–141. 10.1016/S1077-3142(03)00009-2

[B7] Cootes T. F. Taylor C. J. Cooper D. H. Graham J. (1995). Active shape models-their training and application. Comput. Vis. Image Underst. 61, 38–59. 10.1006/cviu.1995.1004

[B8] Duret F. Blouin J.-L. Duret B. (1988). CAD-CAM in dentistry. J. Am. Dent. Assoc. 117, 715–720. 10.14219/jada.archive.1988.0096 3058771

[B9] Huang Z. Yu Y. Xu J. Ni F. Le X. (2020). “PF-net: point fractal network for 3D point cloud completion,” in 2020 IEEE/CVF Conference on Computer Vision and Pattern Recognition (CVPR), Seattle, WA, USA, June 13 2020 to June 19 2020. 10.1109/CVPR42600.2020.00768

[B10] Hwang J.-J. Azernikov S. Efros A. Yu S. (2018). Learning beyond human expertise with generative models for dental restorations. arXiv.

[B11] Iglesias J. E. de Bruijne M. (2007). Semiautomatic segmentation of vertebrae in lateral X-rays using a conditional shape model. Acad. Radiol. 14, 1156–1165. 10.1016/j.acra.2007.06.003 17889333

[B12] Isola P. Zhu J.-Y. Zhou T. Efros A. A. (2017). “Image-to-Image translation with conditional adversarial networks,” in 2017 IEEE Conference on Computer Vision and Pattern Recognition (CVPR), Honolulu, HI, USA, July 21 2017 to July 26 2017, 5967. –5976. 10.1109/CVPR.2017.632

[B13] Jiang X. Dai N. Cheng X. Wang J. Peng Q. Liu H. (2016). Robust tooth surface reconstruction by iterative deformation. Comput. Biol. Med. 68, 90–100. 10.1016/j.compbiomed.2015.11.001 26638148

[B14] Lessard O. Guibault F. Keren J. Cheriet F. (2022). “Dental restoration using a multi-resolution deep learning approach,” in 2022 IEEE 19th International Symposium on Biomedical Imaging (ISBI), Kolkata, India, 28-31 March 2022, 1. –4. 10.1109/ISBI52829.2022.9761622

[B15] Mehl A. Blanz V. Hickel R. (2005). Biogeneric tooth: a new mathematical representation for tooth morphology in lower first molars. Eur. J. Oral Sci. 113, 333–340. 10.1111/j.1600-0722.2005.00224.x 16048526

[B16] Miyazaki T. Hotta Y. Kunii J. Kuriyama S. Tamaki Y. (2009). A review of dental CAD/CAM: current status and future perspectives from 20 years of experience. Dent. Mater. J. 28, 44–56. 10.4012/dmj.28.44 19280967

[B17] Pascoletti G. Aldieri A. Terzini M. Bhattacharya P. Calì M. Zanetti E. M. (2021). Stochastic PCA-based bone models from inverse transform sampling: proof of concept for mandibles and proximal femurs. Appl. Sci. 11 (11), 5204. 10.3390/app11115204

[B18] Peres M. A. Macpherson L. M. D. Weyant R. J. Daly B. Venturelli R. Mathur M. R. (2019). Oral diseases: a global public health challenge. Lancet 394, 249–260. 10.1016/S0140-6736(19)31146-8 31327369

[B19] Song Y.-L. Li J. Yin L. Huang T. Gao P. (2007). The feature-based posterior crown design in a dental CAD/CAM system. Int. J. Adv. Manuf. Technol. 31, 1058–1065. 10.1007/s00170-005-0289-1

[B20] Steinbrecher T. Gerth M. (2008). Dental inlay and onlay construction by iterative laplacian surface editing. Comput. Graph. Forum 27, 1441–1447. 10.1111/j.1467-8659.2008.01284.x

[B21] Susic I. Travar M. Susic M. (2017). The application of CAD/CAM technology in Dentistry. IOP Conf. Ser. Mater. Sci. Eng. 200, 012020. 10.1088/1757-899X/200/1/012020

[B22] Tian S. Wang M. Dai N. Ma H. Li L. Fiorenza L. (2022). DCPR-GAN: dental crown prosthesis restoration using two-stage generative adversarial networks. IEEE J. Biomed. Health Inf. 26, 151–160. 10.1109/JBHI.2021.3119394 34637385

[B23] Wang S. Jiang Y. Hu J. Fan X. Luo Z. Liu Y. (2022). Efficient representation and optimization of TPMS-based porous structures for 3D heat dissipation. Computer-Aided Des. 142, 103123. 10.1016/j.cad.2021.103123

[B24] World Health Organization (2022). Global oral health status report: towards universal health coverage for oral health by 2030: executive summary. Available at: https://www.who.int/publications-detail-redirect/9789240061569 (Accessed February 20, 2023).

[B25] Yuan F. Dai N. Tian S. Zhang B. Sun Y. Yu Q. (2020). Personalized design technique for the dental occlusal surface based on conditional generative adversarial networks. Int. J. Numer. Methods Biomed. Eng. 36, e3321. 10.1002/cnm.3321 32043311

[B26] Zhang C. (2016). Statistical reconstruction algorithm for restoring broken tooth surface based on occlusion spatial constraint. JME 52, 165. 10.3901/JME.2016.01.165

[B27] Zhang C. Liu T. Liao W. Yang T. Jiang L. (2017). Computer-aided design of dental inlay restoration based on dual-factor constrained deformation. Adv. Eng. Softw. 114, 71–84. 10.1016/j.advengsoft.2017.06.005

[B28] Zheng S.-X. Li J. Sun Q.-F. (2011). A novel 3D morphing approach for tooth occlusal surface reconstruction. Computer-Aided Des. 43, 293–302. 10.1016/j.cad.2010.11.003

